# Genomic characterization of rare molecular subclasses of hepatocellular carcinoma

**DOI:** 10.1038/s42003-021-02674-1

**Published:** 2021-10-04

**Authors:** Jeffrey S. Damrauer, Markia A. Smith, Vonn Walter, Aatish Thennavan, Lisle E. Mose, Sara R. Selitsky, Katherine A. Hoadley

**Affiliations:** 1 Lineberger Comprehensive Cancer Center, University of North Carolina at Chapel Hill, Chapel Hill, NC USA; 2 Department of Pathology and Laboratory Medicine, University of North Carolina at Chapel Hill, Chapel Hill, NC USA; 3 Department of Public Health Sciences, Penn State College of Medicine, Hershey, PA USA; 4 Oral and Craniofacial Biomedicine Program, School of Dentistry, University of North Carolina, Chapel Hill, NC USA; 5 Department of Genetics, University of North Carolina, Chapel Hill, NC USA; 6 Computational Medicine Program, University of North Carolina at Chapel Hill, Chapel Hill, NC USA

**Keywords:** Cancer genomics, Hepatocellular carcinoma

## Abstract

Primary liver cancer, consisting of both cholangiocarcinoma (CCA) and hepatocellular carcinoma (HCC), is the second leading cause of cancer deaths worldwide. Our goal is to genomically characterize rare HCC subclasses to provide insight into disease biology. Leveraging The Cancer Genome Atlas (TCGA) to perform a combined analysis of CCA (*n* = 36) and HCC (*n* = 275), we integrated multiple genomic platforms, to assess transcriptional profiles, mutational signatures, and copy number patterns to uncover underlying etiology and linage specific patterns. We identified two molecular classes distinct from prototypical HCC tumors. The first, CCA-Like, although histologically indistinguishable from HCC, had enrichment of CCA mutations (*IDH1*, *BAP1*), mutational signatures, and transcriptional patterns (*SOX9*, *KRT19)*. CCA-Like, however, retained a copy number landscape similar to HCC, suggesting a hepatocellular linage. The second, Blast-Like, is enriched in *TP53* mutations, HBV infection, exposure related mutational signatures and transcriptionally similar to hepatoblasts. Although these subclasses are molecularly distinct, they both have a worse progression-free survival compared to classical HCC tumors, yet are clinically treated the same. The identification of and characterization of CCA-Like and Blast-Like subclasses advance our knowledge of HCC as well as represents an urgent need for the identification of class specific biomarkers and targeted therapy.

## Abstract (web summary)

Jeffrey Damrauer, Markia Smith et al. used existing datasets from cholangiocarcinoma (CCA) and hepatocellular carcinoma (HCC) to characterize two subsets of HCC distinct from prototypical HCC tumors, based on comprehensive analysis of molecular data. The two classes differed from HCC by their copy number, gene expression and mutational signature and exhibited worse progression free survival, highlighting the need to identify class-specific biomarkers and develop targeted therapies for these forms of cancer.

## Introduction

Primary liver cancer is the 2nd and 6th leading cause of cancer death worldwide for men and women, respectively^1^. Within the United States, primary liver cancer rank as the 5th (men) and 7th (women) most deadly cancer^2^. Primary liver cancer includes both hepatocellular carcinoma (HCC) and cholangiocarcinoma (CCA), and although they are anatomically co-localized, they have different etiologic and genomic features^3,4^. HCC is thought to be derived from hepatocytes and accounts for 90% of all primary liver cancer. It has well-characterized risk factors including: chronic hepatitis B/C (HBV and HCV) infection, alcohol abuse, diabetes and aflatoxin exposure^5^. CCA is the second most common primary liver cancer and stems from biliary cells. CCA risk factors include: primary sclerosing cholangitis, hepatobiliary flukes, and biliary tract cysts^6^. Recent publications by The Cancer Genome Atlas (TCGA) identified *IDH1* and *IDH2* mutations, a common feature of CCA, in a subset of HCC samples^7,8^. These *IDH1/2* mutant tumors showed similar gene expression patterns observed in CCA based on ~2,000 genes, as well as displaying similar methylation patterns as other *IDH* mutant tumors. This suggests that HCC tumors may be sub-classified based on their relatedness to CCA. Due to the dearth of targeted treatment options for HCC, further subdividing and characterizing HCC, particularly additional characterization of subset similar to CCA, may aid in the understanding of the disease and, in the future, lead to the identification of new therapeutic targets.

Previous attempts to classify HCC tumors have identified patient populations with gene expression, mutations or survival differences; however, these prior studies were with a limited number of data types, small cohorts or within a singular ancestral or etiologic group^7,9–13^. Our work expands on the previous studies by using a large, harmonized cohort (CCA and HCC), with integrated multi-omic data of samples not restricted to any singular etiology. Through a multi-omic approach, we define three distinct subpopulations of hepatocellular carcinoma tumors, CCA-Like, Blast-Like and HCC. We integrated these subpopulations with external datasets anchoring our data to lineage and cell type-specific cells and indicating a derivation from a hepatocyte lineage.

## Results

### A class of hepatocellular carcinoma tumors shows cholangiocarcinoma gene expression patterns

**Fig. 1:**
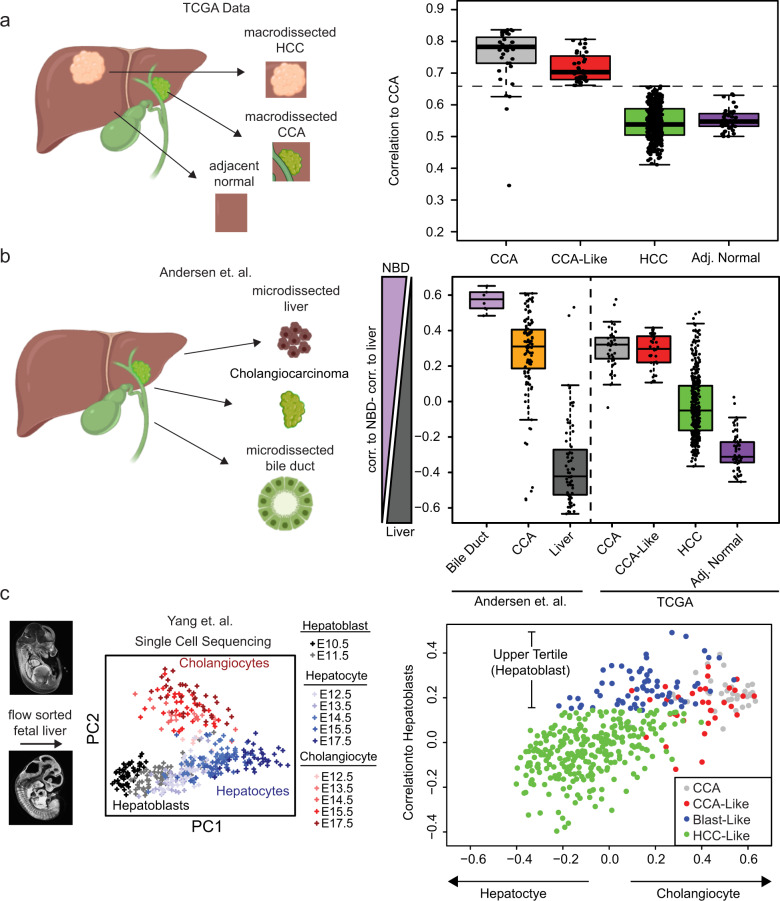
Molecular classification of hepatocellular carcinoma. **a** The Spearman correlation to the median expression of CCA tumors (*n* = 36) was calculated for each TCGA CCA/HCC tumor (*n* = 410). HCC samples within ±1 standard deviation of the mean CCA Spearman correlation (dashed line) were defined at CCA-Like. **b** The CCA/HCC dataset was correlated to microdissected normal bile duct (NBD) (*n* = 6) or normal liver (*n* = 59) from Andersen et al. A NBD vs. Liver score was calculated by subtracting the correlation to normal liver from the correlation to normal bile duct^55^. Boxes represent the IQR with the median represented by the bolded bar. Error bars represent *Q*1/*Q*3 ± 1.5*IQR. **c** Single-cell RNA seq data from Yang et al. was used to correlate the CCA/HCC samples to hepatoblasts (E10.5, *n* = 54), hepatocytes (E17.5, *n* = 34) or cholangiocytes (E17.5, *n* = 34). PCA was performed on Yang et al. to visual variance across the samples then the correlation was calculated between the median expression of day E10.5 and E17.5 (hepatocytes and cholangiocytes) samples to the CCA/HCC dataset. HCC samples in the upper tertile of correlation to hepatoblasts and not prior classified as CCA-Like, were defined as Blast-Like (*n* = 66). Murine embryo images were obtained from http://repo.mouseimaging.ca/repo/4D_embryo_atlases_M_Wong/.

To determine the similarity between TCGA hepatocellular carcinoma samples (HCC) and TCGA cholangiocarcinoma samples (CCA), we calculated each sample’s correlation to a defined CCA centroid (Supplementary Data 1). Thirty-three HCC samples were highly correlated to the CCA centroid (CCA-Like > CCA mean − 1 S.D, mean = 0.75, S.D. = 0.09) and were classified as cholangiocarcinoma-Like (CCA-Like) (Fig. 1a). The rest of the HCC samples had lower correlations to CCA, in a similar range as the tumor-adjacent normal tissues.

As TCGA data comes from bulk specimens, we compared the CCA and HCC cohort to microdissected normal bile duct, normal liver, and cholangiocarcinoma from Andersen et al. (Fig. 1b). The CCA-Like tumors had a higher correlation to normal bile duct than normal liver and in similar range as the CCA samples from TCGA and Andersen cohorts. Whereas HCC samples more closely resembled normal liver, though with a larger range of correlation.

We further anchored the TCGA data to single-cell RNA sequencing data derived from fetal murine livers (embryonic day E10.5−17.5), representing hepatoblasts (E10.5), cholangiocytes (E17.5), and hepatocytes (E17.5) (Fig. 1c). The CCA and CCA-Like samples were correlated to both cholangiocytes and hepatoblasts. While most HCC samples were only correlated to hepatocytes, we also identified a class of HCC samples with high correlation to the hepatoblast cells that had not been previously classified as CCA-Like, these samples were classified as Blast-Like (*n* = 66) (Supplementary Data 1).

### CCA-Like and CCA share genomic alterations

**Fig. 2:**
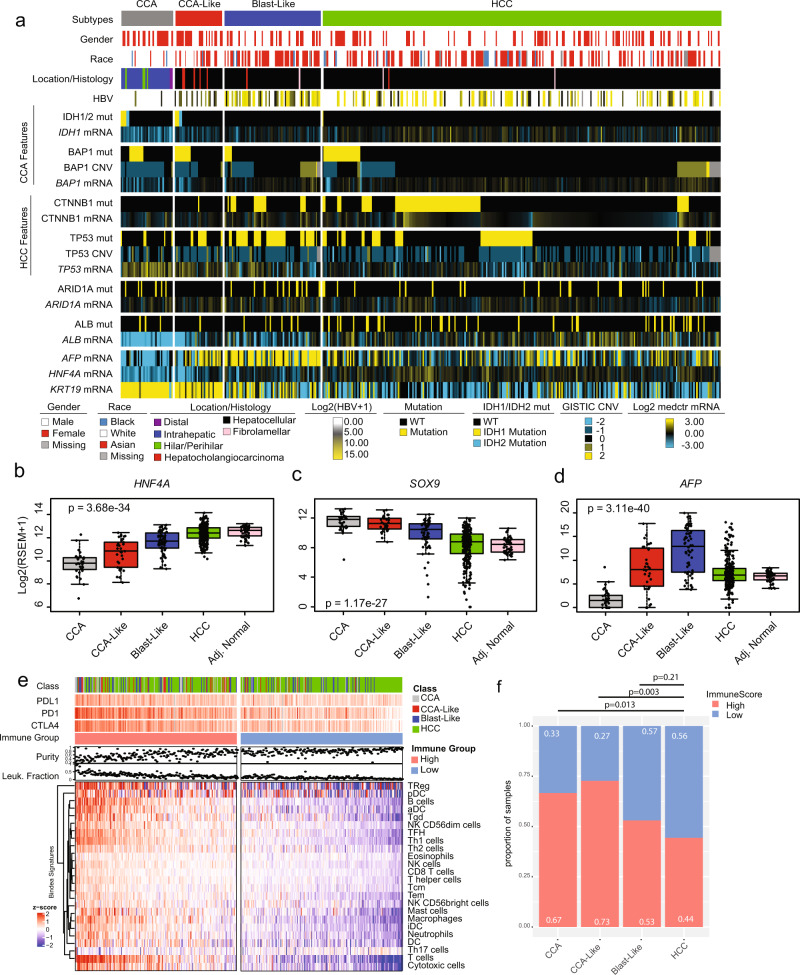
CCA-Like tumors are molecularly similar to CCA. **a** Samples are ordered by subclass and sorted by key genomic alterations. Gene expression data is log2 transformed and median centered. Mutations (mut) are indicated by yellow/blue while wildtype (WT) are indicated by black. GISTIC thresholded values were used for copy number variation (CNV). **b**−**d** Gene expression for hepatocyte markers (*HNF4A and AFP*) and cholangiocyte marker (*SOX9*) are shown per CCA/HCC group. Gene expression values represent the log2 transformed RSEM + 1 value. One-way ANOVA *p*-value is displayed. Boxes represent the IQR with the median represented by the bolded bar. Error bars represent Q1/Q3 ± 1.5*IQR. **e** The median expression across the Bindea immune signatures was calculated and clustered by signatures. Samples were sorted by decreasing median expression across all signature and divided into high and low expression groups. PDL1 [range= 0, 8.5], PD1 [0, 11.1] and CTLA4 [0, 9.9] are represented as Log2(RSEM + 1 values). **f** Stacked bar plots represent the proportion of high and low immune expression group across the tumor classes. Fisher exact test *p*-values are shown for each comparison to HCC.

To further dissect the molecular and clinical characteristics of these tumors, we assessed a series of clinical variables (Supplementary Table 1) as well as mutation, copy number, and gene expression markers of classical alterations in both the CCA and HCC tumors (Supplementary Table 2) (Fig. 2a)^7,8^. As previously described, a subset of TCGA HCC samples had canonical *IDH1/2* mutations (p.R132C/p.R172), a known hallmark of CCA^7^. Interestingly, those mutations were almost exclusively found in the CCA-Like class, except for one HCC tumor with a DNA and RNA variant allele frequency of <0.1 and <0.001% respectively (Supplementary Fig. 1a). Additionally, *IDH1* gene expression was significantly reduced in the CCA-Like tumors compared to the Blast-Like and HCC tumors (Supplementary Table 2). *BAP1* has previously been shown to be frequently altered across both CCA and HCC^7,8^; however, it is almost universally altered in CCA. In the CCA-Like, *BAP1* mutation rate was similar to CCA and copy number levels were lost significantly more than in HCC, yet not to the same degree as CCA (Supplementary Table 2). Compared to HCC, CCA-Like had a decreased mRNA and protein expression of *BAP1* (Supplementary Table 2).

We assessed whether these shared features are due to the CCA-Like class representing a mixed hepatocholangiocarcinoma phenotype. TCGA’s pathology re-review identified only seven cases of hepatocholangiocarcinoma in the TCGA HCC cohort, five of which were in the CCA-Like class, representing 15% of CCA-Like samples (Supplementary Table 1). The remaining CCA-Like samples were unambiguously classified as HCC by histopathology review (Supplementary Fig. 1b−e). A recent study reported that approximately 8% of mixed tumors have *ARID1A* mutations^14^; however, we did not observe any *ARID1A* mutations in the CCA-Like group, while the other classes had mutation frequencies between 8 and 17%.

CCA-Like was almost devoid of the prototypical hepatocellular carcinoma mutations, *CTNNB1* and *TP53*, when compared to Blast-Like, and this group exhibited significantly higher mRNA expression of *p53* when compared to HCC. Interestingly, the Blast-Like class had a significantly higher rate of *TP53* mutation (Fig. 2a), specifically truncating mutations and R249S mutation (Supplementary Fig. 2). As HBV is a risk factor for HCC, we wanted to identify tumors with concurrent HBV infections. Not all samples had the corresponding clinical annotation; therefore, we identified tumors that contained RNAseq reads corresponding to the HBV genome. Blast-Like tumors had increased rates of HBV infection (Supplementary Fig. 3a) as well as a disproportionately high number of patients with Asian ancestry (Supplementary Table 1b) (Fig. 2a). Regardless of tumor class, samples from Asian individuals, had a significantly higher number of HBV mRNA reads (t-test *p*-value = 1.8e−35) compared to the CCA-Like/HCC samples.

### CCA-Like tumors have shared gene-expression features of CCA tumors

The CCA/HCC cohort was clustered using a set of genes associated with either hepatocytes, biliary/progenitor cells, or markers of the cell cycle identified from organoid studies in Hu et al. (Supplementary Fig. 4a)^15^. HCC tumors clustered alongside the tumor-adjacent normal samples and had increased expression for hepatocyte markers such as *ALB* and *HNF4A*. CCA-Like tumors co-clustered with the CCA tumors and similarly had higher expression of the cell cycle and biliary markers but lower expression of hepatocyte markers. CCA had significantly reduced expression of the hepatocyte marker *HNF4A* (*p* = 1.7e−10) (Fig. 2b) and *ALB* (*p* = 1.0e−10) (Supplementary Fig. 4B) and increased expression of cholangiocyte marker *SOX9* (*p* = 3.6e−21) as compared to HCC tumors (Fig. 2c). While the CCA-Like were very similar to the CCA tumors, the CCA-Like cells demonstrated higher gene expression of hepatoblast marker *AFP* (*p* = 8.6e−9) (Fig. 2d)^16^. The Blast-Like tumors had increased expression in cell cycle markers, while displaying an intermediate expression of both biliary and hepatocyte markers, with the exception of *AFP*, which was significantly higher in the Blast-Like tumors compared to all other classes (*p* < 0.001 for all pairwise comparisons (Fig. 2b−d and S2a) (Supplementary Table 2). Additionally, the Blast-Like tumors also had an increased expression-based stemness index, mRNAsi, compared to the other subtypes (Supplementary Fig. 4c)^17^.

We evaluated immune cell patterns across the classes. We visualized the Bindea gene signatures^18^ representing 24 immune cell types and found that samples with high expression of any immune signature were generally high for all immune signatures (Fig. 2e). Samples were grouped by overall median immune gene signature expression and we found the high immune group was associated with a lower tumor purity and an increased DNA methylation leukocyte faction score (Fig. 2e). CAA and CCA-Like classes were enriched with immune signatures and grouped into the immune high set compared to HCC class (Fig. 2f).

### Shared mutational motifs and mutational signatures between CCA and CCA-Like

**Fig. 3:**
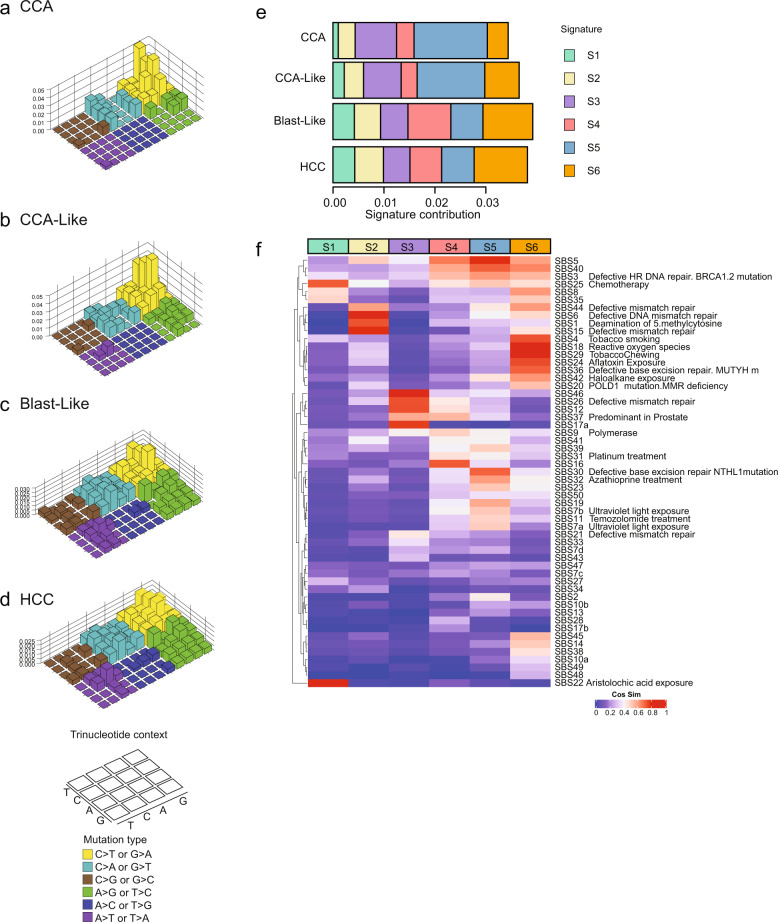
Mutational signatures of CCA and HCC reveal cross-class similarity. **a**−**d** The median frequency of each single nucleotide variant (SNV) per class was calculated and plotted by preceding and succeeding base in a Lego plot (key, bottom left). Base substitutions are divided into six categories to represent the six possible base changes. Substitutions are further divided by the 16 possible flanking nucleotides surrounding the mutated base as listed in the trinucleotide context legend. **e** The R package SomaticSignatures was used to identify *de novo* mutational signatures. Signature contribution across samples is aggregated by class and the median contribution of each signature to the tumor classes is shown. **f** Cosine similarity between COSMIC v3 mutational signatures and each of the *de novo* signatures were computed. The COSMIC signatures are clustered by cosine similarity and ordered by signature class (S1−S6). The color key indicates the degree of similarity.

Reproducible patterns of single nucleotide variants (SNVs), termed ‘mutational signatures’, give a snapshot of the mutational pressures cells have observed, many of which associate with known mutagens^19,20^. We examined our classes to determine if mutational patterns differ by class, particularly in the context of liver cancer which is associated with exposures with known mutational signatures (e.g., aristolochic acid, aflatoxin, and tobacco). CCA-like tumors shared a similar enrichment of C > T/G > T mutations with the CCA tumor class (Fig. 3a, b), while Blast-Like and HCC class tumors shared similar mutational patterns, with decreased C > T/G > T frequency and increased A > T/T > A frequency (Fig. 3c, d). All 96 mutation contexts were hierarchically clustered to visualize the relationships among classes, and were consistent with our transcriptional findings that CCA-like is more related to CCA tumors (Supplementary Fig. 5A). While the top three motifs (all nC > Tn) are shared across the 4 classes, they are most abundant in CCA and CCA-Like. Globally, CCA and CCA-Like also have more diversity of mutation motifs as compared to Blast-Like and HCC (Supplementary Fig. 5a). The per sample motif patterns were then compared against a list of previously discovered single base substitution (SBS) signatures from the COSMICv3 database. The median cosine similarity (CS) for each signature within subclass was calculated and hierarchical clustering was performed and visualized alongside the per sample values. As with the motif level comparisons, CCA and CCA-Like were the most similar with Blast-Like and HCC sharing common feature sets (Supplementary Fig. 5B). We wanted to identify *de novo* mutational patterns. Six mutational signatures (S1-S6) were identified, with S1, S2, and S4 each being primarily driven by a single motif, T > A-CTG, T > C-ATA C > A-GCC, respectively (Supplementary Fig. 5c). Signatures S3 and S5 both were driven by the presence of C > T: GCG, CCG, ACG motifs, with S5 having a low-level increase in a broader range of additional C > T motifs (Supplementary Fig. 5c). The S6 signature, which lacked the presence of any one given motif at a high frequency, had some shared motif patterns with S2 and S5. We quantified the median contribution of each signature for each tumor class and found statistically significant differences between the tumor classes (Fig. 3e and Supplementary Table 4). Signatures S3 and S5 composed the majority of contributions to CCA-Like, a shared feature with CCA, along with having decreased contributions of both S1, S2, and S4 (Fig. 3e). There was no significant difference between CCA and CCA-like for signature S5; however, both classes were significantly enriched for S5 compared to Blast-Like (CCA *p* = 2e−11, CCA-Like *p* = 7e−5) and HCC (CCA *p* = 2e−15, CCA-Like *p* = 2e−6). CCA-Like had an increased prevalence of S6, compared to CCA, a feature shared with the Blast-Like and HCC. Blast-Like and HCC displayed remarkable similarity to each other, with the exception of S4 (*p* = 0.009), which was defining feature of Blast-Like tumors.

To identify possible etiologies of these signatures, we correlated the motif signatures to the COSMICv3 database (Fig. 3f). Signatures S1 and S6 were highly correlated to mutational patterns of chemical or environmental exposures: chemotherapy treatment (S1, SBS25 Cosine Similarity (CS) = 0.72), aristolochic acid (S1, SBS22 CS = 0.96), tobacco (S6, SBS29 CS = 0.83) and aflatoxin (S6, SBS24 CS = 0.74). Defects in mismatch repair patterns defined signature S2 (SBS6 CS = 0.81, SBS15 CS = 0.79) except for signature SBS26, which defined signature S3 (CS = 0.73). Base excision repair (BER) defects dominate signature S5, specifically signatures derived from tumors with *NTHL1* (SBS30, CS = 0.71) and *ERCC2* (SBS5, CS = 0.79) mutations; however, these mutations were not observed within our CCA or CCA-like samples where this signature was enriched. Interestingly, signature S4 defined by SBS16 and enriched in Blast-like currently has an unknown etiology.

Signatures S1 and S6, which contribute most to the liver-specific subtypes (Blast-Like and HCC, and to a lesser extent CCA-Like), have high correlations to known liver carcinogens, aristolochic acid, and tobacco/aflatoxin, respectively. *TP53* mutations, specifically the R249S mutation, have been previously linked to aflatoxin exposure. We observed that tumors with the *TP53* R249S mutation had a significantly higher cosine similarity to the aflatoxin signature (SBS24) than either tumors with alternative *TP53* mutations (*p* = 0.001) or tumors WT for *TP53* (*p* = 3e−4) (Supplementary Fig. 5d), similar to what had been observed in TCGA^7^. The Blast-Like class was dominated by signature S4, which although highly correlated to SBS16 (CS = 0.74), it currently has no known etiology. Conversely, S3 and S5, are shared across CCA and CCA-Like classes, which lack exposure-related correlations, but are enriched for mismatch repair signatures. These results suggest that the mutational pressures in the CCA-like are more similar to CCA and potentially highlighting different selective pressures than the more predominant exposure-based pressures observed in HCC and Blast-Like.

### Transdifferentiation pathways are upregulated in CCA-Like tumors

**Fig. 4:**
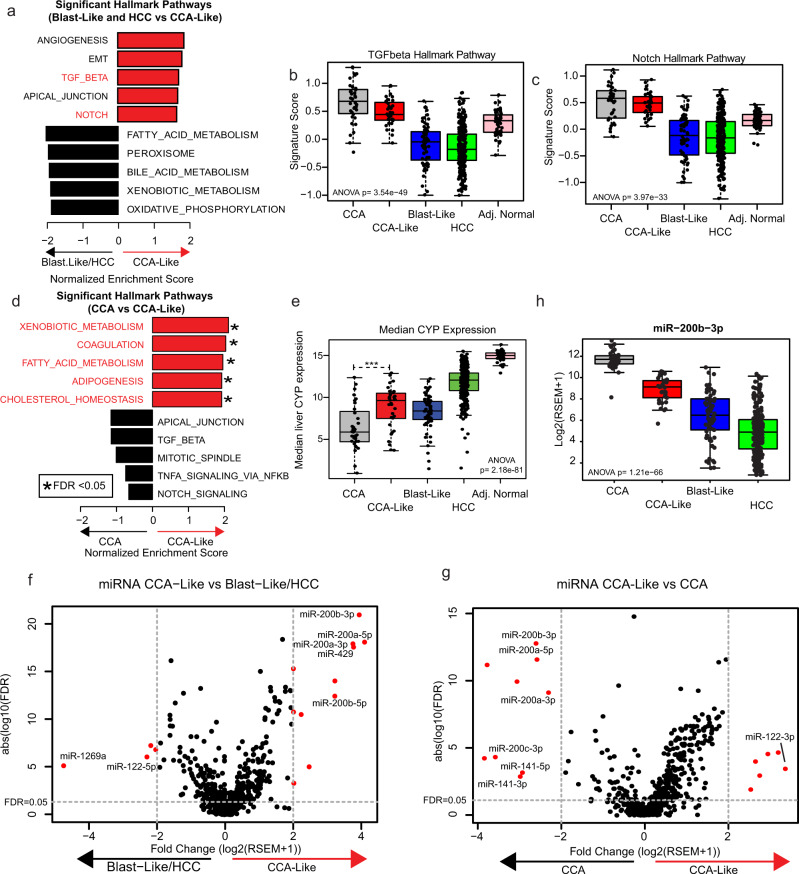
CCA-Like tumors display features of transdifferentiation. **a** Gene Set Enrichment Analysis was performed (CCA-Like vs. Blast-Like and HCC) using the Hallmark gene sets. Seven gene sets were enriched in CCA-Like versus Blast-Like and HCC. Gene sets associated with transdifferentiation are noted in red. **b**, **c** Signature scores associated with transdifferentiation pathways, TGFβ and Notch are plotted by tumor classification. One-way ANOVA *p*-values are shown. **d** Gene set enrichment analysis comparing CCA-Like vs. CCA using the hallmark gene sets. Liver-specific gene sets are noted in red. **e** The median expression of liver-specific cytochrome P450 genes are plotted by subtype, *** indicates *p* < 0.001 for two-sample t-test between CCA and CCA-Like. **f**−**g** Volcano plots for fold change vs FDR; are plotted for CCA-Like vs. Blast-Like and HCC and CCA-Like vs. CCA respectively; genes with fold change > 2 and FDR < 0.05 are indicated in red. **h** Per class expression of miR-200b-3p, a representative family member of the miR-200 family is plotted, *p*-value represents ANOVA. Boxes represent the IQR with the median represented by the bolded bar. Error bars represent Q1/Q3 ± 1.5*IQR.

We explored the genomic/transcriptomic features driving each tumor class to determine if there are signaling pathways that are shared across classes. We performed gene set enrichment analysis (GSEA) using the hallmark gene signature list to identify differential pathway signatures between CCA-Like and the other HCC tumor classes (Blast-Like and HCC) (Fig. 4a)^21,22^. Of the seven significant gene sets (nominal *p*-value <0.05 and FDR < 0.25), three pathways, TGFβ, NOTCH, and WNT have previously been implicated as drivers of Epithelial-Mesenchymal Transition (EMT) and transdifferentiation in the liver (Supplementary Fig. 6a−c)^23–29^. TGFβ, NOTCH, and WNT expression signatures were all significantly elevated within the CCA-Like tumors compared to the HCC classes (CCA-like vs HCC *p* < 0.001) (Fig. 4b, c and S6d). For these three signatures, the CCA-Like tumors module scores were not significantly different to those observed in CCA albeit slightly reduced (TGFβ *p* = 0.015, NOTCH *p* = 0.70, WNT *p* = 0.62). We further compared the CCA-Like tumors directly to CCA samples. Four of the top five significantly enriched pathways in CCA-Like tumors related to liver biology (Fig. 4D). We plotted the median expression of 10 Cytochrome P450 (CYP) genes that are abundant in the liver as a surrogate for liver-specific gene expression and found that CCA-Like tumors had significantly higher levels of expression of CYP genes compared to CCA tumors (t-test, *p* = 3.23e−5). CCA-Like tumors expressed CYP at comparable levels to the Blast-Like tumors which were still lower than what was observed in HCC or adjacent normal liver tissue (Fig. 4e).

Pathway level analysis identified multiple pathways that are involved in both transdifferentiation, as well as EMT. miRNA expression has been shown to drive both processes. We performed a differential miRNA expression analysis and identified four mir-200 family members highly enriched in both CCA and CCA-Like tumors (Fig. 4f, g) compared to Blast-Like and HCC. Blast-Like and HCC samples had increased expression of miR-122, a liver-specific miRNA^30^. Interestingly, when CCA-Like and CCA are directly compared, miR-122 was the most enriched miRNA in the CCA-like, while the miR-200 family members were significantly enriched in the CCA (FC = 3.7, FDR < 0.001). Expression of one representative miR-220 family member, miR-200b-3p, was highest in CCA, followed by CCA-like and lowest in HCC (Fig. 4h).

### The CCA-Like copy number landscape resembles that of HCC

Although the CCA-Like class tumors bear a striking transcriptional resemblance to the CCA class, key markers (elevated *AFP/ALB* and miR-122) indicate that the CCA-like tumors still have features shared with HCC and suggests the precursor cell likely arises from hepatocytes rather than a cholangiocyte/hepatocyte progenitor cell. Because copy number alterations are often considered early events in transformation^31^, we compared the copy number landscapes of CCA, CCA-Like, Blast-Like, and HCC to infer the shared cellular origin of the classes.

**Fig. 5:**
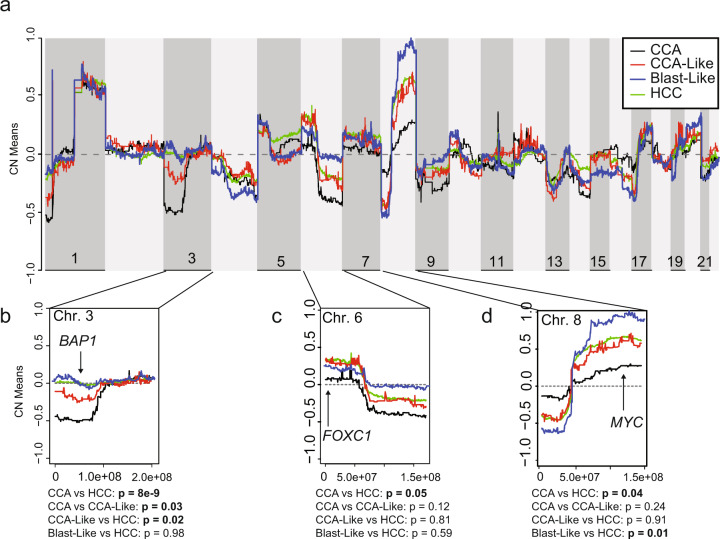
Copy number landscapes show tissue-specific specificity. **a** Genome-wide copy number values are plotted for CCA (black), CCA-Like (red), Blast-Like (blue), and HCC (green) using the mean quantitative gene-level copy measurements from GISTIC. **b**−**d** Expanded view of three chromosomes containing regions significantly differed by pairwise two-sample t-tests (*p*-value <0.05) between CCA and HCC. Potential target genes in significant segments are noted.

Overall, CCA-Like, Blast-Like, and HCC displayed a more similar copy number landscape to each other than to CCA (Fig. 5a). Using SwitchDNA, we performed a pairwise comparison of segments comparing CCA-Like to CCA and HCC, and Blast-Like to CCA and HCC. CCA-Like had significantly fewer copy number differences with HCC (*n* = 360) than CCA (*n* = 1024) (*p* < 0.0001) (Supplementary Fig. 7a). There were 272 segments that were significant in CCA-Like in both comparisons to CCA and HCC, all of which (except one segment) were between 3p24.3−12.3. Located within this region is *BAP1*, which is almost universally lost in CCA. *BAP1* was lost to a lesser extent in CCA-Like (45% of samples) than CCA (80% of samples); however, at a significantly greater frequency than HCC (12%) (*p* < 0.05) (Supplementary Table 2 and Fig. 5b). Conversely, *FOXC1* (6p25.3) and *MYC* (8q24.21) are amplified in the CCA-Like, Blast-Like, and HCC tumors (Fig. 5c, d). The CCA-Like and Blast-Like classes displayed numerous differences in copy number landscape frequency compared to CCA and HCC (Fig. 5a). Blast-Like tumors had increased genomic instability, which resulted in a more distinct copy number landscapes, 1143 segments were significantly different as compared to HCC and 3489 segments as compared to CCA with 2690 segments shared as significantly different compared to both CCA and HCC (Supplementary Fig. 7b). The gene expression was compared to the GISTIC copy number values for *BAP1*, *FOXC1,* and *MYC*. Decreased *BAP1* gene expression was correlated to a decreased to the copy number status for all classes (across all samples, *p*-value = 2.2e−16, CCA, CCA-Like, Blast-Like, and HCC *p* < 0.001) (Supplementary Fig. 7c). Although *MYC* was amplified in CCA-Like, Blast-Like, and HCC, only in the Blast-Like and HCC classes was gene expression and copy number correlated (Blast-Like and HCC *p*-value < 1.0e−4), whereas gene expression and copy number were not correlated in any class for *FOXC1* (Supplementary Fig. 7d, e).

### CCA-Like and Blast-Like have decreased progression-free survival

**Fig. 6:**
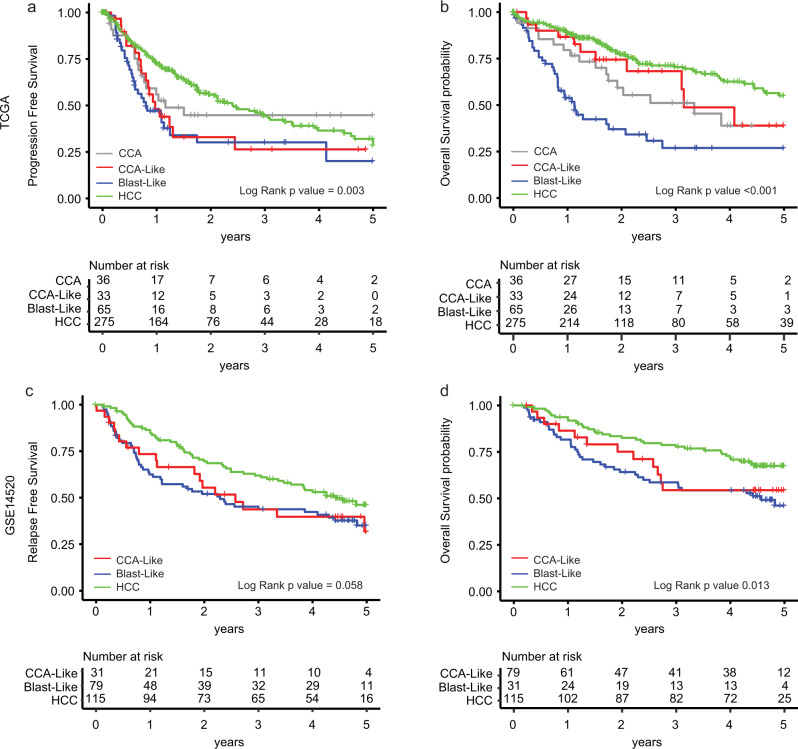
Blast-Like tumor have worse progression-free and overall survival. Kaplan−Meier curves of TCGA CCA/HCC data for **a** progression-free and **b** overall survival. Tumor classifications were predicted on to GSE14520 using ClaNC for **c** relapse-free and **d** overall survival^32^. All survival data was censored at 5 year and log-rank *p* value was calculated.

Progression-free and overall survival censored at five years was compared across classes (Fig. 6a, b) (Supplementary Table 3). Using a Cox proportional hazards model, Blast-Like tumors had significantly worse progression-free (Hazard Ratio [HR] 1.95, *p* < 0.001) and overall (HR 3.72, *p* < 0.001) survival comparted to HCC. CCA-Like tumors had worse progression-free survival compared to HCC (HR 1.68, *p*-value = 0.04), but not overall survival. We also looked at models including the clinical factors stage and grade. In a univariate model, only stage was associated with outcomes. When we added stage to the model with our subclasses, only the Blast-like class retained significance for worse progression-free and overall survival compared to the referent class HCC. AFP protein expression has additionally been shown to be a prognostic marker, as such, we performed a multivariate analysis; when adding *AFP* gene expression to the model, Blast-Like and CCA-Like were still significant predictors of worse progression-free (HR = 1.9, *p* = 0.002 and HR = 1.6, *p* = 0.03, respectively) and Blast-Like was significant predictor of worse overall survival (HR = 3.5, *p* = 8e−10). We generated a gene expression classifier for our classes based on TCGA data and applied it to Roessler et al. HCC cohort as a validation dataset (GSE14520)^32^. The five-year survival within the validation cohort displayed similar trends to TCGA. The Blast-Like class trended towards a worse relapse-free survival (HR 1.4, *p*-value = 0.055) and had significantly worse overall survival with and without adjusting for stage (HR 1.9, *p*-value = 0.006, HR 2.0, *p*-value = 0.002, respectively) (Fig. 6c, d and Table 3).

We next applied the Hoshida et al. and Woo et al. signatures to our TCGA dataset using consensus clustering. Hoshida identified three subclasses (S1, S2, and S3) that were correlated to clinical and molecular features^10^. Woo et al. identified a cholangio-like group of tumors (CLCHCC) and then further divided the subtypes with respect to stemness^9^. With both the Hoshida and Woo subtyping strategies, the CCA-Like and Blast-Like samples were grouped together within the S2 and CLCHCC classes respectively (Supplementary Fig. 8a−c). The stem cell signature from Ben-Porath et al.^33^ was applied accordingly to the Woo et al. dataset and the CLCHCC class was further divided into stem cell signature positive or negative (Supplementary Fig. 8c). Overall, all groups identify subtypes with similar, but not completely overlapping features. Our scheme helps solidify the classifications by anchoring with true CCA and incorporating microdissected and single-cell data.

Progression-free and overall survival curves were generated using TCGA data for both our classes as well as Hoshida and Woo classifications (Supplementary Fig. 8d−i). For progression-free survival, Blast-like and CCA-Like had the shortest time to progression with a median time of 301 days and 355 days, respectively, as compared to HCC (879 days) (*p* = 1.2e−9, *p* = 0.01) (Supplementary Fig. 8D). Within the Hoshida subtypes, no significant difference in survival was observed (Supplementary Fig. 8e). The median time to progression for the poorest outcome CLCHCC (ES) class was 355 days (*p* = 2.0e−4) as compared to CLCHCC (neg ES) and HCC (neg ES), which had a median time to progression >700 days (Supplementary Fig. 8g). Our Blast-like classification identified a subset of patients with poorer outcomes.

## Discussion

In this study, we used a harmonized TCGA CCA/HCC dataset to characterize HCC samples based on their similarity to CCA and the precursor hepatoblast cell type. Previous studies have noted similar transcriptional classes^7–9^. Here, we have expanded on these classifications and used a multi-omic approach, as well as using external data to characterize rare molecular subtypes in an integrated manner; the result of which is the identification of three distinct classes of HCC tumors: CCA-Like, Blast-Like, and HCC.

Previous work had identified *IDH1* mutant and CCA-like subclasses of tumors similar to the aforementioned CCA-Like. Our work is a natural extension of the groundwork laid in these papers. Woo et al. identified a cholangiocarcinoma-like group with varying levels of embryonic stem (ES) cell marker expression. Whereas the expression of ES signatures led them to conclude that these tumors were derived from a bi-potent progenitor cell, hepatoblast; our current study, with the addition of copy number data, demonstrates that the CCA-like group has a copy number landscape that more closely resembles HCC. This in combination with the expression of liver-specific genes, albeit at reduced levels, is an indication that the CCA-like class is more likely to be derived from hepatocytes that were transformed and underwent dedifferentiation and initiated a transdifferentiation transcriptional program in response to specific alterations (e.g., *BAP1*, *IDH1/2*). The Cancer Genome Atlas Project previously described *IDH1/2* mutant HCC and presented evidence that these tumors overlapped CCA via a TumorMap visualization, that incorporated DNA, DNA methylation, and expression features; however, this group was restricted to a small subset with *IDH1/2* mutation. Our work expanded this group to include a set of transcriptionally similar tumors that includes samples with a high frequency of *IDH1*/2 mutations and alterations in *BAP1*, as well as linking this subclass to the induction of a transdifferentiation program.

A majority of the tumors originating from liver displayed classical HCC features including expression of *ALB* and *HNF4A* genes as well as mutations in *CTNNB1* (29%). However, Blast-like tumors exhibited more frequent mutations in *TP53* (58%) and elevated *AFP* expression, a hepatoblast marker gene. It has been previously reported that *TP53* mutations, specifically R249S mutations, are most commonly observed in east Asian populations and are associated with aflatoxin exposure^34^. Corroborating this R249S/Aflatoxin relationship, we saw that tumors with the R249S mutations also had high similarity to a previously described Aflatoxin mutational signature (Supplementary Fig. 5d). Furthermore, the Blast-like class was enriched with patients of Asian ancestry (Supplementary Table 1) as well as having a significantly higher rate of HBV infection (Supplementary Tables 1 and S4) (Fig. 2a and Supplementary Fig. 3b). The association between Asian ancestry and HBV + HCC is expected, as the historic prevalence of HBV infection in East Asia is ~7.3% as compared to the North America at 0.3%^35^. Additionally, many of the samples of Asian ancestry within the TCGA come from Asian tissue source site. Current data has linked chronic HBV infection to immune induced liver injury^36–38^. This injury can result in dedifferentiation of hepatocytes^39^ and in turn, based on our data, may lead to a more hepatoblast-like disease in east Asian populations.

CCA-Like lacks prototypical mutations and risk factors associated with HCC. Hirsch et al. previously described a *BAP1* mutant class of tumors that lacked *CTNNB1* mutation and canonical risk factor but has similarity to fibrolamellar tumors^40^. Our work builds on this group as CCA-Like tumors are enriched for *IDH1/2* and *BAP1* mutations and have transcriptional patterns similar to CCA. By using transcriptional patterns to define the CCA-Like class, we found that in addition to *BAP1* mutations, *BAP1* copy number loss was another frequent mechanism for decreasing *BAP1* mRNA expression levels (Supplementary Fig. 7c).

By performing mutational signature profiling, we observed the CCA-Like had a more similar global mutational signature profile to CCA than HCC. However, CCA-Like still showed underlying exposure-based signatures though their overall contribution was reduced compared to HCC (Fig. 3e, f). A hepatocellular cell-of-origin is reinforced by the observation that the CCA-Like and HCC tumors have similar expression levels of *ALB* and *AFP*. The CCA-Like tumors were also classified as hepatocellular carcinomas by TCGA’s expert pathology re-review. Woo et al. has identified a similar subclass (Cholangiocarcinoma-Like), but as previously mentioned these authors attribute this to the transformation of a progenitor hepatocellular cell vs a hepatocyte^9^. However, the CCA-Like tumors have increased *ALB* expression and liver-specific gene and miRNA expression signatures when compared to CCA, suggesting linkage to hepatocytes. More recently Wardell et al. demonstrated that genomic features of intrahepatic CCA tumor suggest a hepatocyte cell of origin^41^. While our work is an agreement with that notion, we extend their findings through transcriptional analysis proposing upregulation of transdifferentiation pathways. We show that CCA-Like have upregulation of NOTCH, WNT, and TGFβ pathways as compared to Blast-like and HCC, all of which are known to be associated with transdifferentiation. A murine study by Sekiya and Suzuki demonstrated that NOTCH signaling in hepatocytes can induce the conversion of hepatocytes to biliary cells leading to the development of cholangiocarcinoma^25^. Additionally, in NOTCH deficient mice, expression of *TGFB* allows the generation of the biliary tree from hepatocytes^24^.

This notion of transdifferentiation is bolstered by the finding that the copy number landscape of CCA-Like is more similar to HCC than CCA (Fig. 5a). Copy number alterations are thought to be early events in tumor development; this data corroborates the mRNA data by suggesting CCA-Like is derived from a hepatocyte rather than a bipotent progenitor cell. Additionally, the CCA-specific DNA alterations, *IDH1/2* and *BAP1*, could be driving this transdifferentiation process in the absence of classical HCC mutations such as *CTNNB1* and *TP53*. Artegiani et al. recently reported that *BAP1*−/− organoids upregulated *EPCAM* while downregulating liver-specific genes, consistent with our findings (Supplementary Fig. 4a)^42^.

Prior groups have laid a strong basis for subtype classification including the identification of subtypes similar to the CCA-like^8–10,40^ and Blast-like^10,12,13^. We have added to this rich body of work with a direct comparison to true CCA and incorporated mutational signature analysis and copy number data to further describe the underlying biology and potential cell of origin for these classes. Our findings link a specific class of HCC tumors with transdifferentiation; however, further work will need to be done to validate this mechanistically to identify the genomic alterations and signaling pathway changes that are necessary and sufficient to drive the CCA-Like tumor type.

One limitation of our study was the partial availability of TCGA’s non-required data elements including: serum markers, family histories, consistently annotated risk factors and long-term follow-up. More in-depth and standardized annotation for these clinical data elements will be critical to better understand associations between the molecular data and etiologic risk factors.

Through the integration of multiple data types, we were able to expand on prior work, which identified a subset of HCC tumors that resembled CCA. We identified three distinct classes of hepatocellular carcinoma, in which the CCA-Like class may be derived through the initiation of a transdifferentiation process, rather than transformation of progenitor cell.

## Methods

### Tumor classification

Upper quartile normalized RSEM gene expression data for TCGA was downloaded from the GDC legacy archive (https://portal.gdc.cancer.gov/legacy-archive/). Cholangiocarcinoma (CCA dataset, *n* = 36) and Hepatocellular carcinoma (HCC dataset, *n* = 374) samples from TCGA were merged, log2 transformed, and filtered for highly expressed and variably expressed genes (*n* = 4035). The data was median centered across genes. The Spearman correlation to the median gene expression of all CCA samples was calculated to determine the per sample similarity for all CCA and HCC samples. Samples within (-) 1 standard deviation from the mean correlation of all CCA samples were classified as CCA-Like. To determine similarity to hepatoblast cells, we used single-cell RNAseq data of hepatoblast differentiation in mice (GSE90047)^43^. HCC samples were correlated to variably expressed genes from hepatoblasts (E10.5) and differentiated hepatocytes (17.5, DLK^+^, EPCAM_-_) and cholangiocytes (17.5, DLK^-^, EPCAM^+^). HCC samples with a correlation to hepatoblasts in the upper tertile of all samples and not otherwise classified as CCA-Like were classified as Blast-Like. The resulting classification yielded, CCA (*n* = 36), CCA-Like (*n* = 33), Blast-Like (*n* = 66), HCC (*n* = 275).

### mRNA analysis

All external datasets were log2 transformed and median centered across genes. The Spearman correlation value was calculated between the CCA/HCC samples and the median expression of the comparison classes (CCA, HCC, NBD, Liver, Cholangiocytes, Hepatocytes, and Hepatoblasts). To compare the CCA/HCC dataset to microdissected normal bile duct and normal liver, the TCGA dataset was merged with GSE26566 by adjusting TCGA data to the median expression of GSE26566 cholangiocarcinoma samples. The Spearman correlation was calculated as described above comparing the CCA and HCC cohort to microdissected normal bile duct, normal liver, and cholangiocarcinoma. To generate a differentiation score, the per sample correlation to Normal Liver was subtracted from that sample’s correlation to NBD. This was also done for correlations to hepatocytes and Cholangiocytes. Hepatitis B virus was detected in CCA and HCC tumors RNAseq data via VirDetect^44^. To visualize gene expression patterns across known markers of cholangiocytes and hepatocytes, CCA/HCC samples were hierarchically clustered (Cluster3.0), using expression markers from Hu et al.^15^. Gene Set Enrichment (GSEA) was performed one vs rest comparisons across classes for all HCC tumors. Significance was determined using a nominal *p*-value <0.05 and FDR < 0.25.

Hoshida and Woo subtypes were derived by extracting the respective gene signatures and performing ConsensusClusterPlus to determine the expression groups. Hierarchical clustering was performed using the ES1 signature from Ben-Porath et al., the samples within the increased gene expression cluster were selected as ES1 enriched^33^.

Variant calling on RNA-Seq data was performed by aligning RNASeq reads with STAR^45^ in two pass mode with unmapped reads assigned to the mate’s position when possible. Parameters outFilterScoreMinOverLread and outFilterScoreMinOverLread were set to 0.45. Reads were realigned using ABRA2^46^. Reads were sorted and duplicates were marked using biobambam^47^ both before and after running ABRA2. Indels were called using Cadabra.

All analysis was performed in R (Version 3.5.2) unless otherwise noted.

### miRNA

*miRNA* RSEM data was downloaded from the GDC legacy archive (https://portal.gdc.cancer.gov/legacy-archive/) and log2 transformed. To determine significantly differentially expressed miRNAs, t-tests were performed on a per gene basis, CCA-Like vs. Blast-Like/HCC and CCA-Like vs. CCA. Benjamini−Hochberg adjusted *p*-values were calculated to account for multiple comparisons.

### Genomic features

Copy number and mutation data were downloaded from FireBrowse (http://gdac.broadinstitute.org). Lollipop plots were generated through cBioPortal.org^48,49^. Additional *BAP1* alterations were determined using the de novo aligner ABRA2^46^. Chi-square and Fisher’s exact test were performed when appropriate in a pairwise manner. For mRNA expression, a two-sample t-test was performed to determine the significance of expression with CCA/HCC classification classes. The biomaRt R package was used to identify genomic positions for genes. Custom R scripts based on functions in the MVisAGe R package^50^ were used to plot mean gene-level DNA copy number values in each expression subtype as well as differences of mean gene-level DNA copy number values between pairs of gene expression subtypes.

To identify disease class-specific copy number alteration (CNA) we used SWITCHplus (https://genome.unc.edu/SWITCHplus/)^51^. Fisher’s exact test was performed between paired comparison classes to identify class-specific CNAs. Segment’s significance was assigned using Benjamini−Hochberg adjusted *p*-values <0.05.

### Characterizing mutational signatures in LIHC and CHOL

The R package SomaticSignatures was used to identify mutational signatures in 396 TCGA HCC and CHOL whole-exome sequencing samples^52^. Motif contributions across the samples were aggregated by RNA class. The R package barplot3d was used to generate 3D barplots displaying the frequency of the 96 different combinations of somatic mutations and trinucleotide contexts seen in this cohort. Using COSMIC mutational signatures version 3, we performed cosine similarity (CS) between our six signatures and the 49 SBS signatures to further characterize the mechanisms underlying our signatures. Liver sample motifs were correlated with our signatures to determine similarities and whether signatures are subtype-specific, as we previously saw with the correlation to COSMIC mutational signatures v3.

### Class prediction, survival analysis, and clinical variables

Using the CCA/HCC as the training dataset, a gene classifier (*n* = 150) was generated using ClaNC (Supplementary Data 2)^53^. Predictions were made on GSE14520 using the correlation to the training set centroids, and Kaplan−Meier curves were generated using the survminer package in R. Univariate Cox proportional hazards models were used to determine the significance of the tumor classes, stage, and grade. Variables significant in the univariate analysis were then incorporated into a multivariate model. Clinical data for TCGA was obtained from Liu et al.^54^. Clinical data that was included in the analysis had >94% of data present across the cohort (race, gender, age, stage, grade, and survival). As part of TCGA, diagnostic and frozen slides were reviewed by a panel of pathologists with expertize in hepatobiliary cancers. The panels consisted of six pathologists for HCC^7^ and five pathologists for CCA^8^.

### Statistics and reproducibility

Categorical variables were compared using Fisher’s exact or chi-square test. Continuous variable comparisons were made using t-test or ANOVA as indicated. Correlations were performed using Pearson or Spearman correlation as indicated. Multiple comparison correction was performed using Bonferroni correction. Survival analyses were performed using Kaplan−Meier with log-rank tests. Statistical analyses were performed using R unless otherwise noted.

### Reporting summary

Further information on research design is available in the Nature Research Reporting Summary linked to this article.

## Supplementary information

Supplementary Information

Description of Supplementary Files

Supplementary Data 1

Supplementary Data 2

Reporting Summary

## Data Availability

TCGA data is available through the gdc data portal, https://portal.gdc.cancer.gov/. Expression data is available by download from https://www.ncbi.nlm.nih.gov/geo/ accession number: GSE14520 (Roessler et al.), GSE90047 (Yang et al.), GSE26566 (Andersen et al.). All underlying data for figures are available: 10.6084/m9.figshare.15180810.v1

## Code availability

All code is available from the corresponding author upon reasonable request.
