# Supplementary material for: Genomic characterization of rare molecular subclasses of hepatocellular carcinoma
Source: Commun Biol. 2021 Oct 4;4:1150. doi: 10.1038/s42003-021-02674-1 (PMC8490450; doi:10.1038/s42003-021-02674-1)
Supplement: Supplementary file 1 — Supplementary Information [file 42003_2021_2674_MOESM1_ESM.pdf]

a

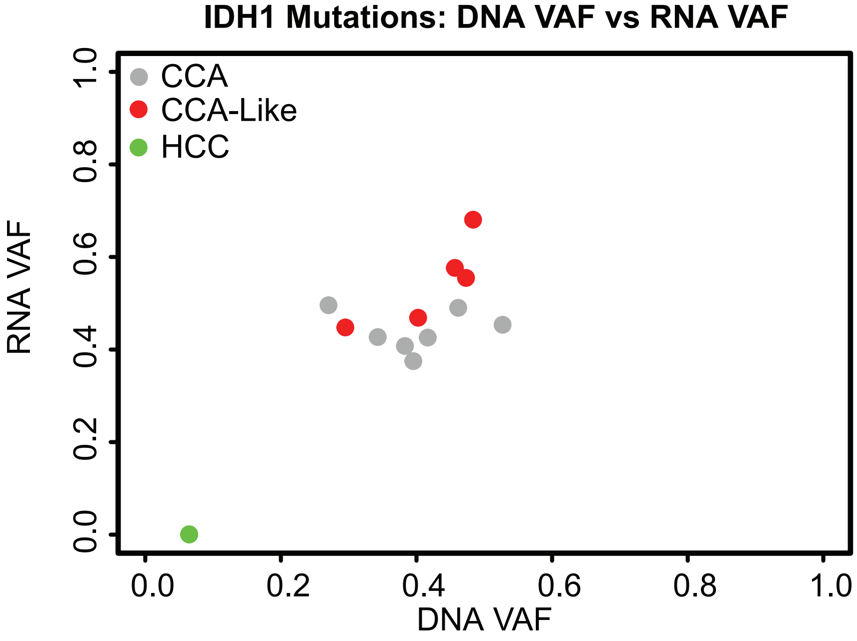

b. CCA: TCGA-W6-AA0S

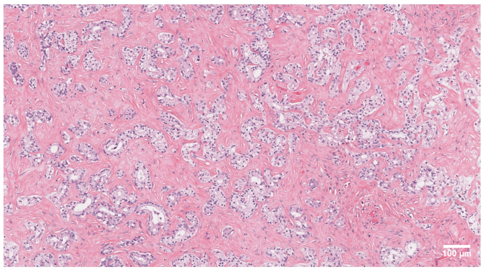

d. Blast-Like: TCGA-DD-A1EL

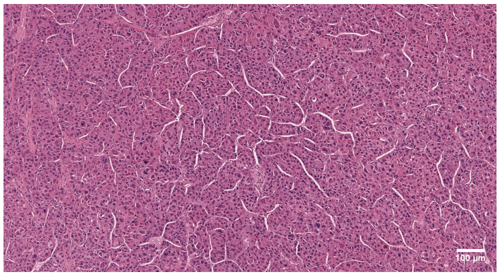

c. CCA-Like: TCGA-5C-A9VH

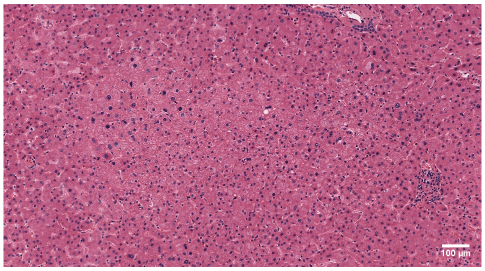

e. HCC: TCGA-NI-A8LF

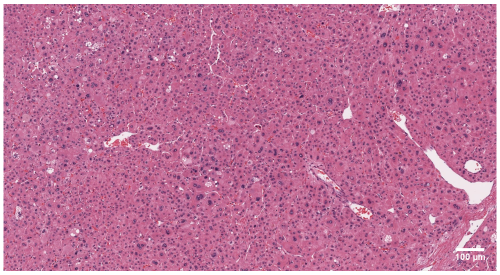

Supplementary Figure 1 - (a) Variant allele frequencies for IDH1 were calculated from both the DNA and RNA sequencing reads. Tumor classes are indicated by color (grey=CCA, red=CCA-Like, green=H-CC). (b) Representative H&E slides (20x) of CCA (c) CCA-Like (d) Blast-Like (e) HCC. (https://cancer.digitalslidearchive.org/).

Supplementary Figure 2

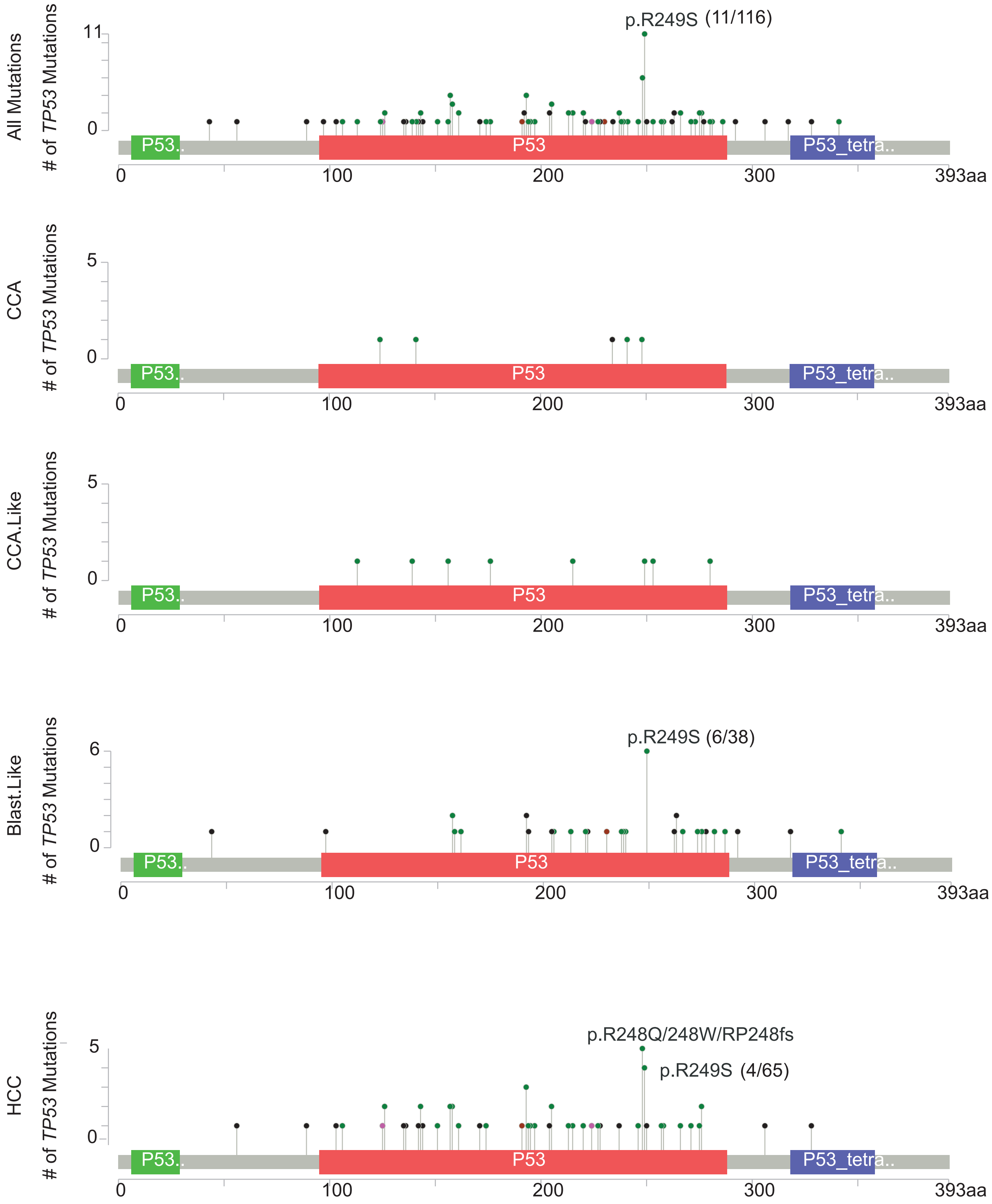

Supplementary Figure 2 – Lollipop plots for TP53 mutations are shown for the entire cohort and by tumor class. R249S mutation ratios are shown as (number of R249S mutations)/ (total number of TP53 mutations) per group.

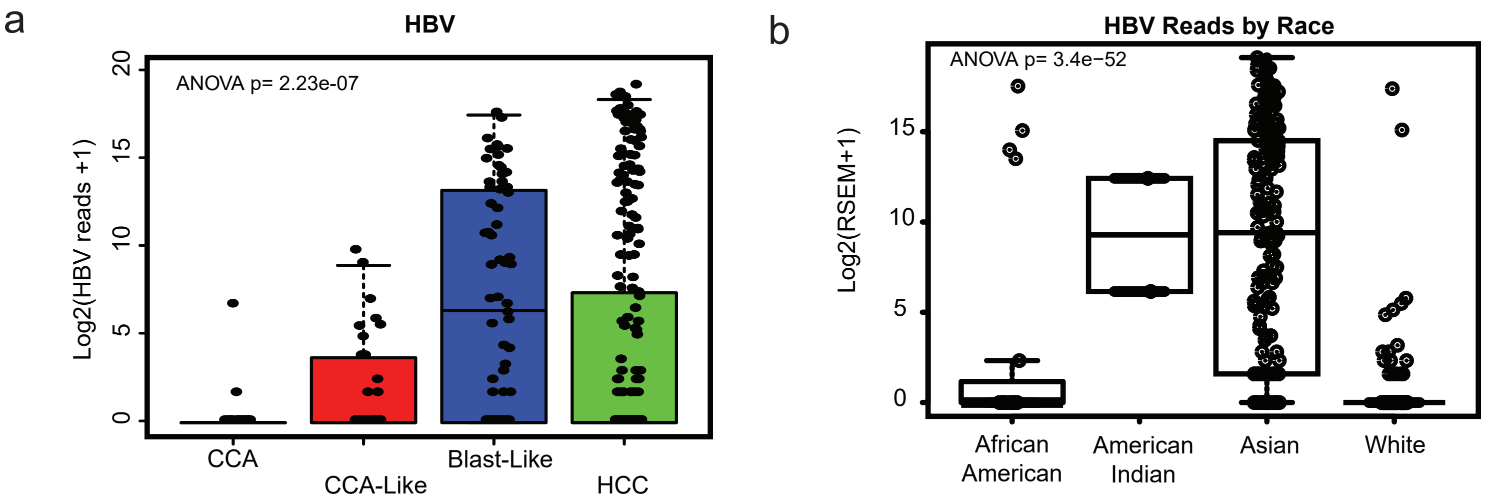

Supplementary Figure 3 - (a) Reads aligning to HBV are plotted by tumor class and (b) reported race

Supplementary Figure 4

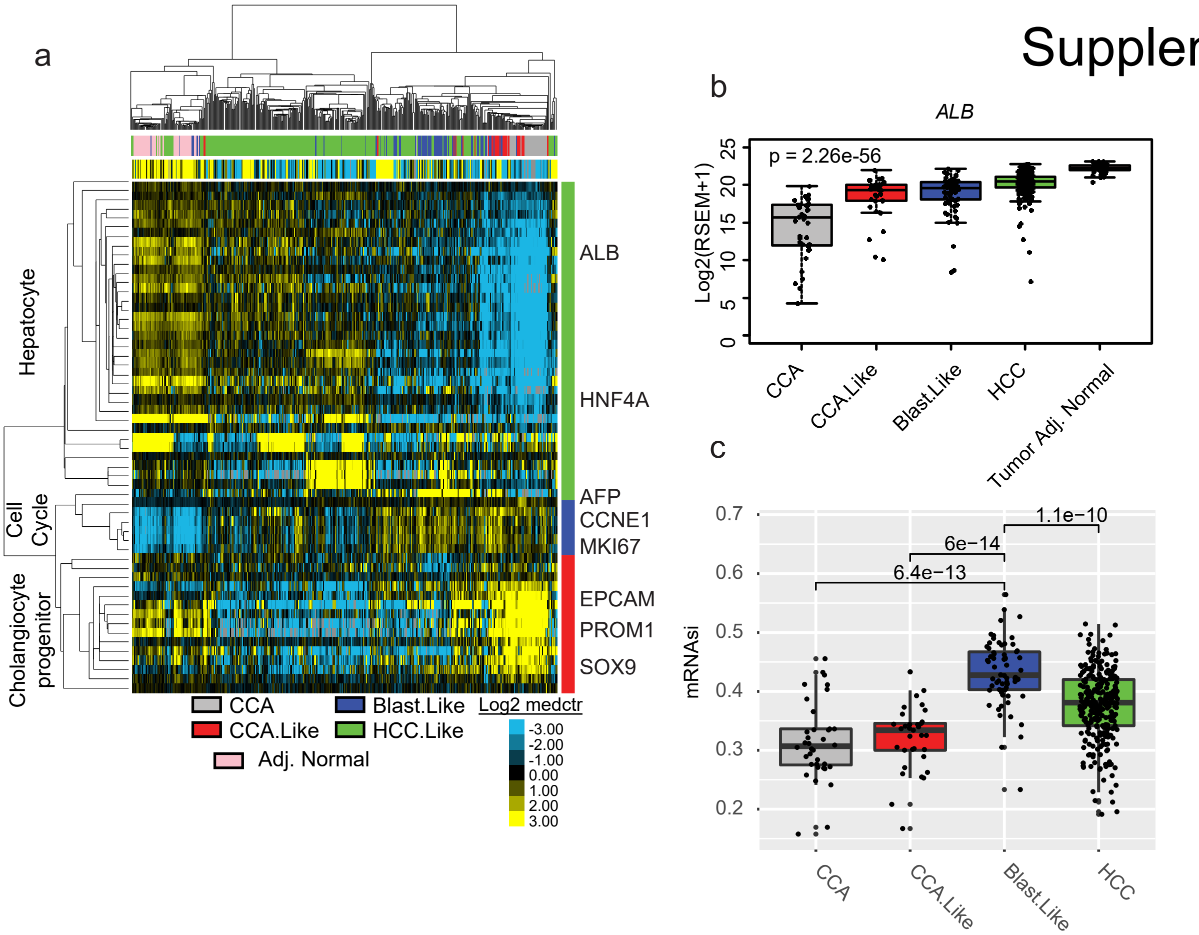

Supplementary Figure 4 – (a) CCA/HCC tumors and adjacent normal tissues clustered with cell type specific markers of hepatocytes and cholangiocytes cells and cell cycle markers from Hu et. al.[13.] Gene expression data was log2 transformed and median centered across the CCA/HCC cohort. Annotation bar represents sample subclasses. (b) Gene expression for *ALB* is shown per CCA/HCC group (ANOVA p=2.26e-56). Gene expression values represent the log2 transformed RSEM+1 value. (c) The expression based stemness index from Malta et. al. was plotted by class. p-values represents t-test.

Supplementary Figure 5

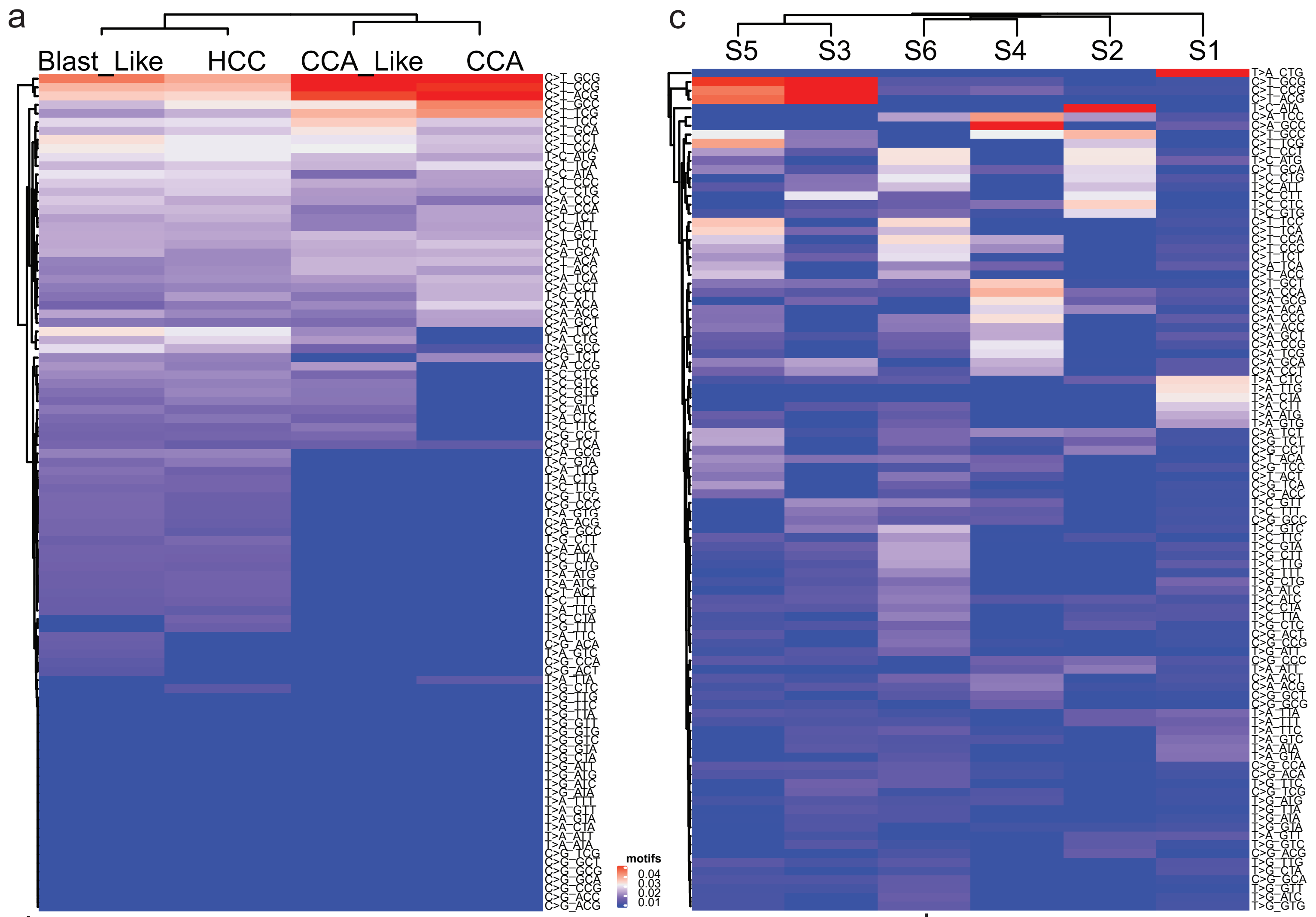

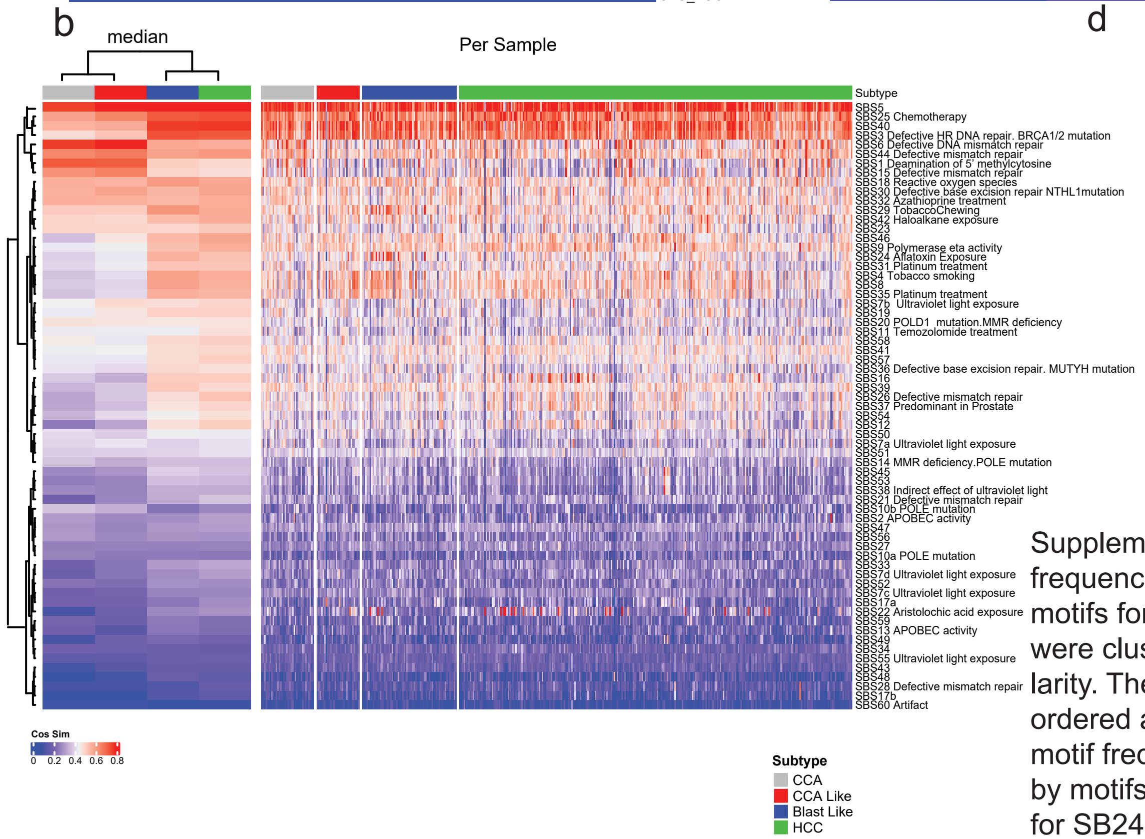

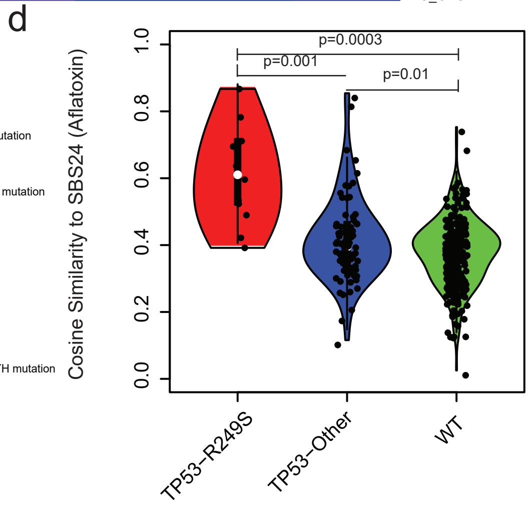

Supplementary Figure 5 – The median motif frequency was hierarchically clustered by motifs for (a) classes. (b)COSMIC signatures were clustered by median class cosine similarity. The per sample CS was then similarity ordered and sorted by class. (c) The median motif frequency was hierarchically clustered by motifs for signatures. (d) Cosine similarity for SB24 (aflatoxin exposure) was plotted by TP53 mutation type (TP53 R249S, Others or WT) and pairwise t-test were performed to determine significance.

Supplementary Figure 6

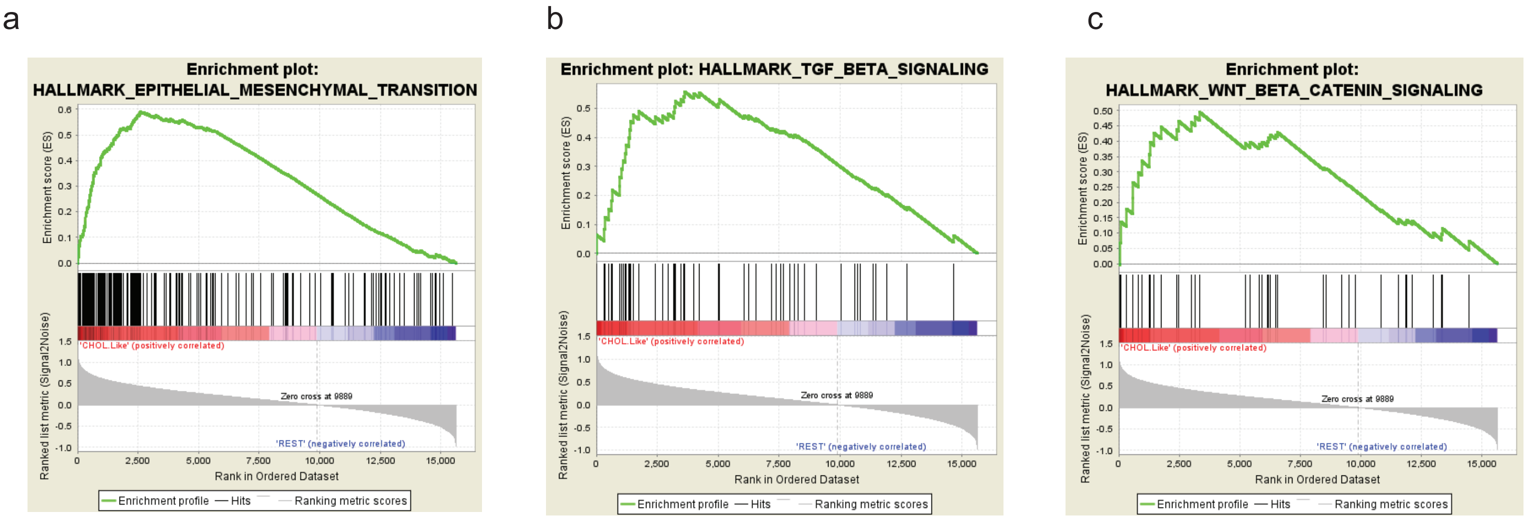

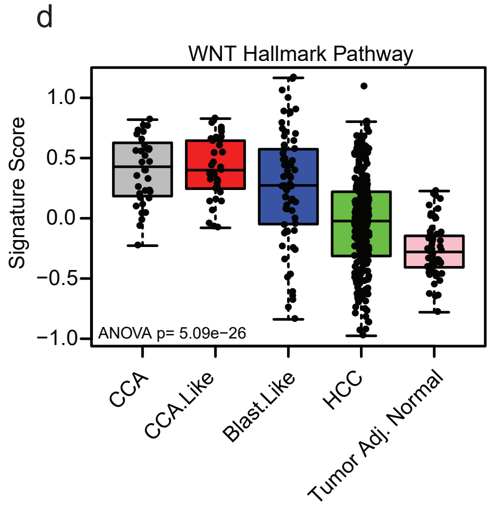

Supplementary Figure 6 - (a-c) GSEA was performed comparing CCA-Like vs. Blast-Like/HCC. Plots of enrichment scores are shown for pathways relating to transdifferentiation. (d) Signature score associated with WNT pathway are plotted by class.

Supplementary Figure 7

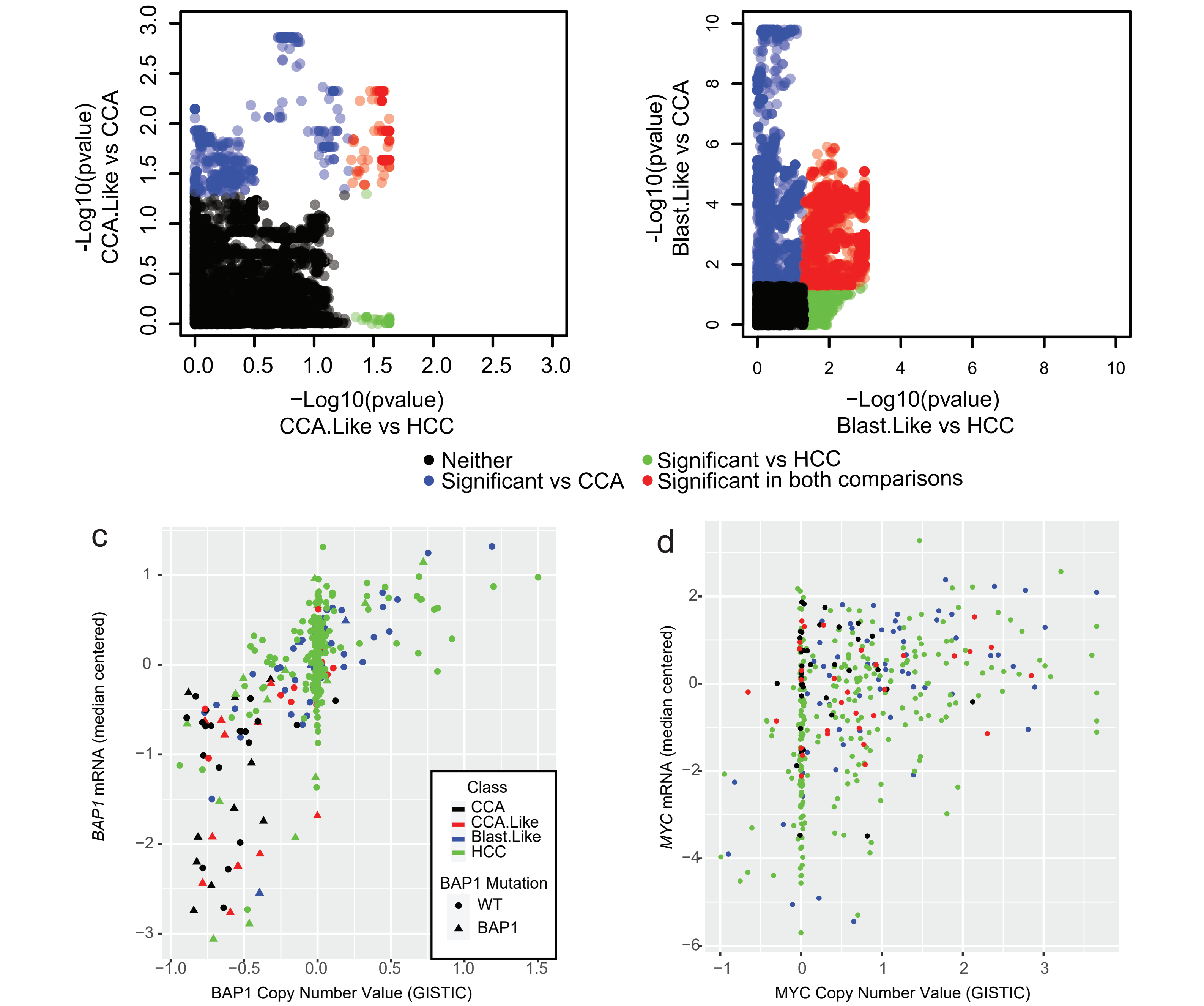

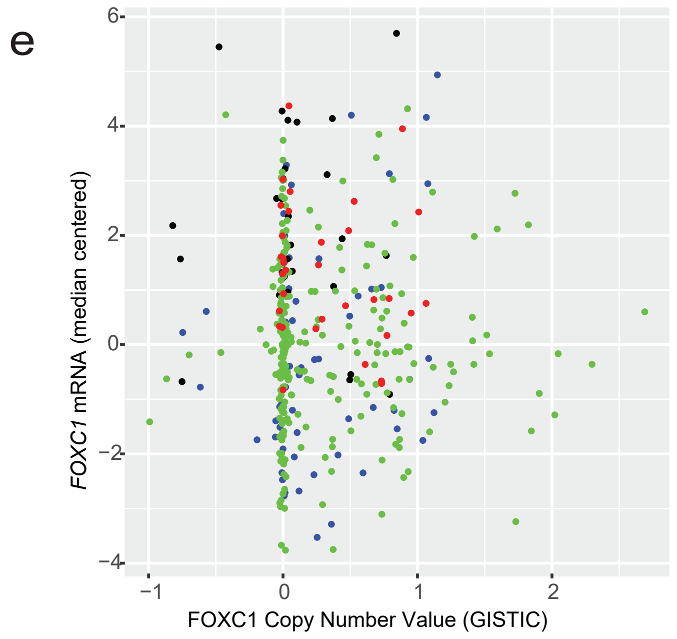

Supplementary Figure 7- (a) Pairwise two sample t-tests were performed by segment and p-values were corrected for multiple comparisons. –log10 (adjusted pvalue) was plotted for CCA-Like vs CCA and HCC and (b) Blast-Like vs. CCA and HCC. Log2 median centered gene expression was plotted against the GISTIC intensity values for (c) BAP1, (d) MYC and (e) FOXC1. For BAP1 mutations noted by symbols.

Supplementary Figure 8

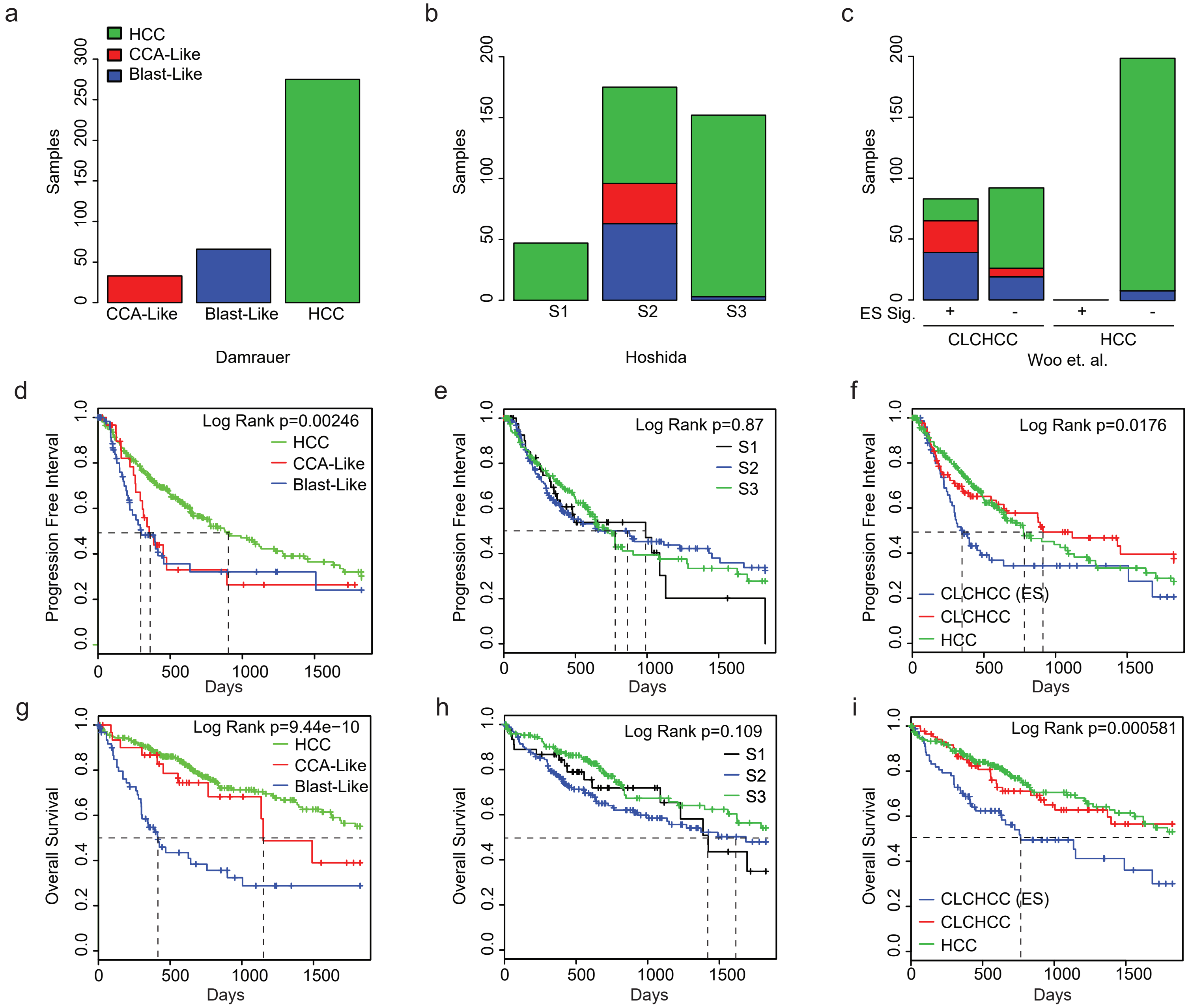

Supplementary Figure 8– (a-c) Hoshida et. al. and Woo et. al. subtype classifications were applied to TCGA cohorts. (d-f) Progression-free and (g-i) overall survival were plotted for the given tumor classifications. Dashed lines represent 50% survival.

Supplemental Table 1. Clinical Characteristics of TCGA CCA and HCC datasets

	CCA	CCA-like	Blast-like	HCC
No. of Cases				
	36	33	66	272*
Gender				
Female	20 (56%)	20 (61%)[b,c]	21 (32%)	80 (29%)
Male	16 (44%)	13 (39%)	45 (68%)	192 (71%)
Race				
African American	2 (6%)	1 (3%)	3 (5%)	13 (5%)
Asian	3 (8%)	12 (36%)	40 (60%)[e]	106 (39%)
White	31 (86%)	20 (61%)	22 (33%)	142 (52%)
Other/NA	0 (0%)	0 (0%)	1 (2%)	11 (4%)
Stage				
I	19 (53%)[b]	12 (36%)	18 (27%)	141 (52%)[a,b]
II	9 (25%)	7 (21%)	22 (33%)	57 (21%)
III	1 (3%)	10 (30%)	24 (36%)	51 (19%)
IV	7 (19%)	0 (0%)	1 (2%)	4 (1%)
NA	0 (0%)	4 (12%)	1 (2%)	19 (7%)
Grade				
G1	1 (3%)	1 (3%)	5 (8%)	49 (18%)[e]
G2	15 (42%)	14 (42%)	20 (30%)[c]	143 (53%)
G3	18 (50%)	14 (42%)	36 (54%)[c]	72 (26%)
G4	2 (5%)	3 (9%)[c]	5 (8%)[c]	4 (1%)
NA	0 (0%)	1 (3%)	0 (0%)	4 (1%)
ECOG Score				
0	20 (56%)	14 (42%)	17 (26%)[c,d]	131 (48%)
1	9 (25%)	5 (15%)	10 (15%)	69 (25%)
2	0 (0%)[b]	1 (3%)	11 (17%)[c,d]	14 (5%)
3	1 (3%)	1 (3%)	6 (9%)[d]	5 (2%)
4	0 (0%)	0 (0%)	3 (5%) [d]	0 (0%)
NA	6 (17%)	12 (36%)	19 (29%)	53 (19%)
Histology				
Fibrolamellar	0 (0%)	0 (0%)	1 (2%)	2 (1%)
Hepatocellular	0 (0%)	28 (85%)	64 (97%)	269 (99%)
Hepatocholangiocarcinoma	0 (0%)[a]	5 (15%)[e]	1 (2%)	1 (0%)
HBV infection [f]				
Positive	2 (6%)	12 (36%)	44 (67%)[e]	97 (36%)

Negative	34 (94%)	21 (64%)	22 (33%)	174 (64%)
NA	0 (0%)	0 (0%)	0 (0%)	4 (1%)
Cirrhosis/Fibrosis				
No Fibrosis	16 (44%)[e]	5 (15%)	7 (11%)	62 (23%)
Fibrosis	11 (31%)	7 (21%)	5 (8%)[d]	47 (17%)
Cirrhosis	0 (0%)[e]	5 (15%)	10 (15%)	64 (24%) [d]
NA	9 (25%)	16 (48%)	44 (67%)	99 (36%)

[a] Significant as compared to CAA.Like ($p<0.05$)

[b] Significant as compared to Blast.Like ($p<0.05$)

[c] Significant as compared to HCC ($p<0.05$)

[d] Significant as compared to CAA ($p<0.05$)

[e] Significant as compared to all other groups ($p<0.05$)

[f] HBV infection determined by >5 HBV reads from RNA-seq

*272/275 samples had unique clinical annotation

Supplemental Table 2. Molecular Characteristics of TCGA CCA and HCC datasets

	CCA	CCA-like	Blast-like	HCC
No. of Cases				
	36	33	66	275
IDH				
IDH1/IDH2 mutation	6	5[b,c]	0	1
IDH1 mRNA (median)[f]	11.63[e]	12.43[e]	12.84	13.29
BAP1				
BAP1 mutation	10 (27%)[b,c]	11 (33%)[b,c]	5 (8%)	25 (9%)
BAP1 copy number loss	29 (80%)[e]	15 (45%)[c,d]	17 (26%)	33 (12%)
BAP1 mRNA (median)[f]	10.3	10.73	11.07	11.12
CTNNB1				
CTNNB1 mutation	0 (0%)	1 (3%)	18 (27%)[a,d]	79 (29%)[a,d]
CTNNB1 mRNA (median)[f]	11.84[b,c]	12.43[b,c]	12.6	12.44
TP53				
TP53 mutation	5 (14%)	8 (27%)	38 (58%)[e]	65 (24%)
TP53 copy number loss	13 (36%)[e]	23 (70%)	45 (68%)	152 (55%)
TP53 mRNA (median)[f]	10.83[e]	10.28[e]	9.86	9.55
ARID1A				
ARID1A mutation	6 (17%)	0 (0%)[e]	11 (17%)	23 (8%)
ARID1A mRNA (median)[f]	10.43	10.68[c]	10.56	10.4
ALB				
ALB mutation	3 (8%)	1 (3%)	6 (9%)	36 (13%)
ALB mRNA (median)[f]	15.65	19.34	19.58	20.44
AFP				
AFP mRNA (median)[f]	1.48	8.06[d]	12.97[e]	6.88[d]
HNF4A				
HNF4A mRNA (median)[f]	9.80[e]	10.84[e]	11.69	12.42
KRT19				
KRT19 mRNA (median)[f]	13.02[e]	11.78[e]	7.04	4.04

[a] Significant as compared to CAA.Like ($p<0.05$)

[b] Significant as compared to Blast.Like ($p<0.05$)

[c] Significant as compared to HCC ($p<0.05$)

[d] Significant as compared to CAA ($p<0.05$)

[e] Significant as compared to all other groups ($p<0.05$)

[f] mRNA expression is represented as Log2 median-centered values

Supplemental Table 3. Univariate and multivariate Cox regression analysis

Univariate analysis				
TCGA	Progression Free Interval	Progression Free Interval	Overall Survival	Overall Survival
Subtype (HCC ref)	Hazard Ratio (95% CI)	p – value	Hazard Ratio (95% CI)	p – value
CCA	1.22 (0.73 - 2.02)	0.448	1.90 (1.12 -3.23)	**0.017**
CCA-Like	1.68 (1.02 - 2.76)	**0.04**	1.45 (0.77 – 2.73)	0.245
Blast.Like	1.95 (1.32 – 2.88)	**<0.001**	3.72 (2.48 – 5.56)	**<0.001**
Stage (I ref)				
II	1.96 (1.38 – 2.77)	**<0.001**	1.63 (1.06 – 2.50)	**0.027**
III	2.35 (1.63 – 3.40)	**<0.001**	2.56 (1.69 – 3.87)	**<0.001**
IV	4.28 (2.20 – 8.35)	**<0.001**	5.26 (2.65 – 10.47)	**<0.001**
Grade (G1 ref)				
G2	1.44 (0.89 – 2.32)	0.133	1.34 (0.76 – 2.35)	0.316
G3	1.55 (0.95 – 2.52)	0.078	1.39 (0.78 – 2.48)	0.259
G4	1.51 (0.61 – 3.75)	0.775	2.46 (1.00 – 6.06)	**0.05**
Multivariate analysis				
Subtype + Stage (HCC ref)				
CCA	1.31 (0.79 – 2.18)	0.301	2.06 (1.21 – 3.51)	**<0.01**
CCA-Like	1.57 (0.95 – 2.59)	0.078	1.39 (0.73 – 2.63)	0.318
Blast.Like	1.70 (1.14 – 2.54)	**<0.01**	3.43 (2.28 – 5.17)	**<0.001**
Subtype + Stage +Grade (HCC ref)				
CCA	1.25 (0.72 – 2.11)	0.403	2.12 (1.22 – 3.68)	**<0.01**
CCA-Like	1.15 (0.85 – 2.46)	0.171	1.27 (0.64 – 2.50)	0.496
Blast-Like	1.62 (1.07 – 2.46)	**0.023**	3.30 (2.14 – 5.09)	**<0.001**
GSE14520				
Subtype (HCC ref)				
CCA-Like	1.49 (0.88 – 2.54)	0.142	1.60 (0.83 – 3.10)	0.161
Blast-Like	1.56 (1.06 – 2.30)	**0.025**	1.97 (1.24 – 3.14)	**0.004**
Stage (I ref)				
II	1.99 (1.30 – 3.05)	**<0.01**	2.07 (1.19 – 3.61)	**0.01**
III	3.17 (1.98 – 5.08)	**<0.001**	5.10 (2.92 – 8.93)	**<0.001**
Multivariate analysis				
Subtype + Stage (HCC ref)				
CCA-Like	1.1 (0.63 – 1.91)	0.745	1.11 (0.56 – 2.18)	0.77
Blast-Like	1.730 (0.87 – 1.95)	0.199	1.60 (.99 – 2.59)	0.054

Supplemental Table 4 - Bonferroni corrected pvalues of pairwise t-test between mutational signatures and subclasses

	**S1**	**S2**	**S3**	**S4**	**S5**	**S6**
**CCA vs CCA-Like**	1	1	1	1	0.26	0.95285
**CCA vs Blast-Like**	**0.002**	1	0.078	**0.00039**	**2.10E-11**	**0.00031**
**CCA vs HCC**	**0.0024**	1	0.093	0.16226	**2.10E-15**	6.40E-**09**
**CCA-Like vs Blast-Like**	0.0696	1	0.055	0.06847	**7.40E-05**	0.15424
**CCA-Like vs HCC**	0.1356	0.11	0.067	1	**2.40E-06**	0.**00075**
**Blast-Like vs HCC**	1	1	1	0.**00854**	1	0.39735

Bolded values denote p<0.05
