# Supplementary material for: Genomic characterization of rare molecular subclasses of hepatocellular carcinoma
Source: Commun Biol. 2021 Oct 4;4:1150. doi: 10.1038/s42003-021-02674-1 (PMC8490450; doi:10.1038/s42003-021-02674-1)
Supplement: Supplementary file 2 — Description of Supplementary Files [file 42003_2021_2674_MOESM2_ESM.pdf]

Description of Supplementary Files

File name: Supplementary data 1
Description: Per Sample subclass calls with the corresponding correlations to CCA and Blast centroids.

File name: Supplementary data 2
Description: 150 gene classifier with corresponding subgroup centroid values.
